# Supplementary material for: Patterns of Response to Immune Checkpoint Inhibitors in Association with Genomic and Clinical Features in Patients with Head and Neck Squamous Cell Carcinoma (HNSCC)
Source: Cancers (Basel). 2021 Jan 14;13(2):286. doi: 10.3390/cancers13020286 (PMC7828787; doi:10.3390/cancers13020286)
Supplement: Supplementary file 1 [file cancers-13-00286-s001.pdf]

Supplementary Materials:

Patterns of Response to Immune Checkpoint Inhibitors in Association with Genomic and Clinical Features in Patients with Head and Neck Squamous Cell Carcinoma (HNSCC)

Panagiota Economopoulou, Maria Anastasiou, George Papaxoinis, Nikolaos Spathas, Aris Spathis, Nikolaos Oikonomopoulos, Ioannis Kotsantis, Onoufrios Tsavaris, Maria Gkotzamanidou, Niki Gavrielatou, Elena Vagia, Efthymios Kyrodimos, Eleni Gagari, Evangelos Giotakis, Alexander Delides and Amanda Psyrri

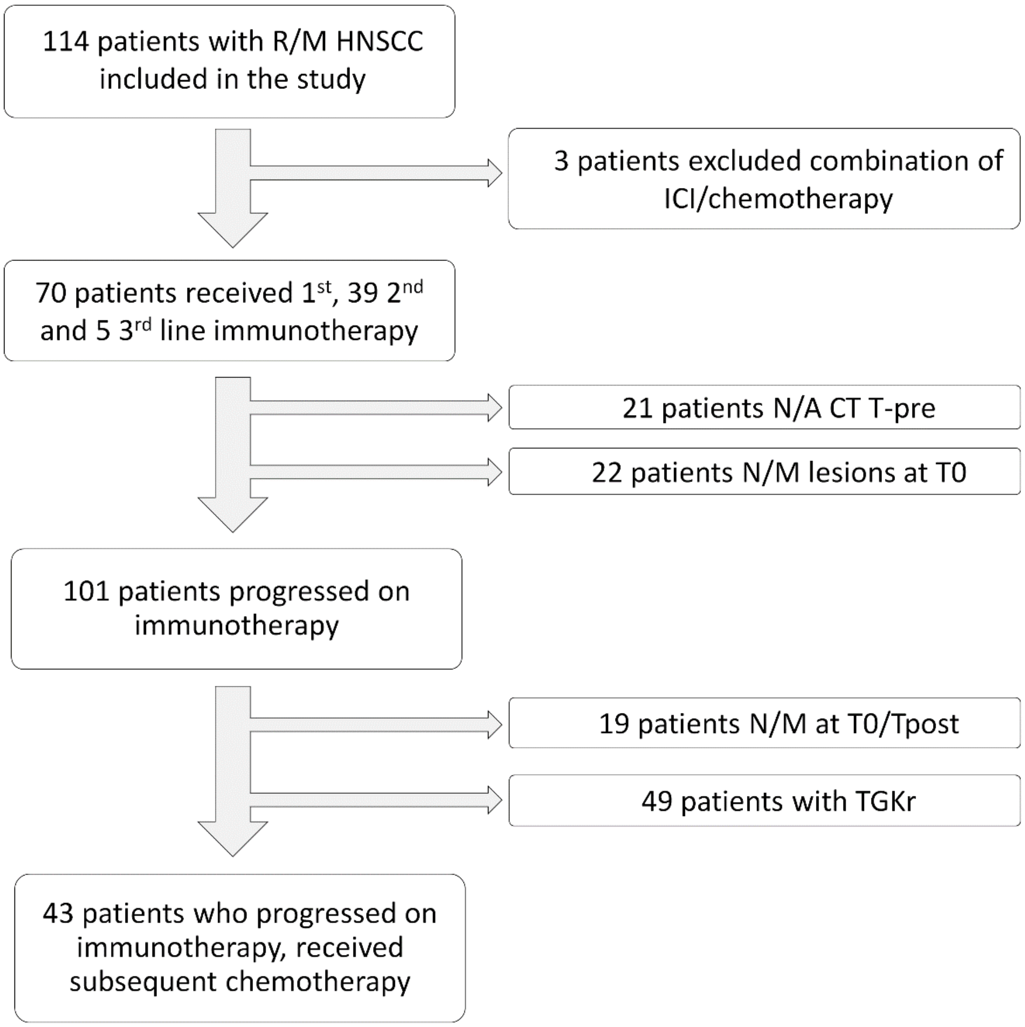

Figure S1. REMARK diagram of the study.

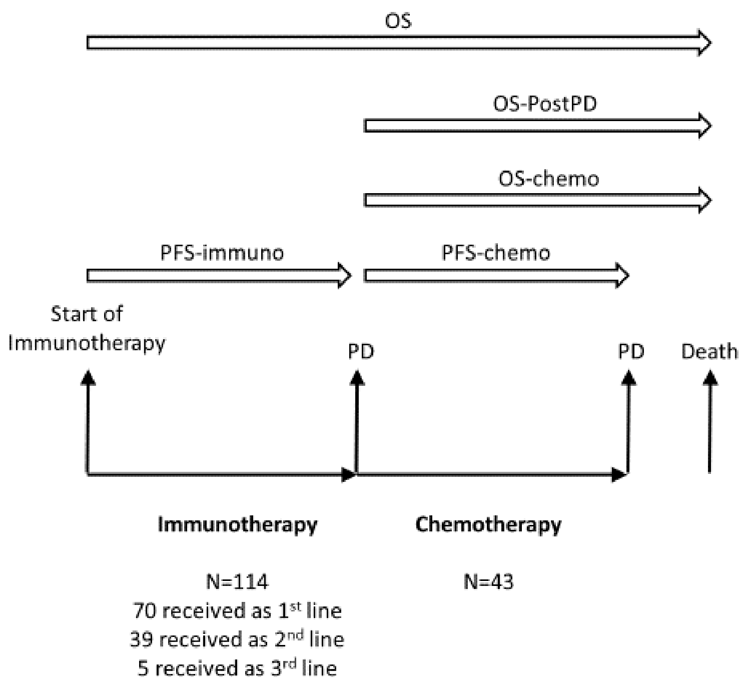

Figure S2. Graphical description of survival time definitions.

Table S1. Basic characteristics of patients. Statistical significance is noted in bold.

Characteristic		*N*	%
Age	<65 years	32	65.3
	≥65 years	17	34.7
Sex	Male	40	81.6
	Female	9	18.4
History of tobacco use	No	7	14.9
	Light/Median	5	8.6
	Heavy	35	74.5
	Unknown	2	4
History of alcohol consumption	No	19	41.3
	Social	10	21.7
	Heavy	14	30.4
	Unknown	4	8.2
Primary tumor site	Oral cavity (including lip)	22	44.9
	Oropharynx/Hypopharynx	5	10.2
	Larynx	17	34.7
	Nose/paranasal sinuses	5	10.2
Disease setting	Metastatic	23	46.9
	Recurrent	26	53.1

Table S2. Association of basic characteristics with type of TGK ratio progression.

Characteristic			Type of Progressive Disease		*p*-Value
			Non-HPD	HPD	
Age (*N* = 49)		Median (95%CI)	65.1 (59.8–67.3)	53.8 (47.1–61.5)	**0.036**
Age	<65 years	*N* (%)	18 (58.1.0%)	14 (77.8%)	0.219
	≥65 years	*N* (%)	13 (41.9%)	4 (22.2%)	
Sex	Male	*N* (%)	26 (83.9%)	14 (77.8%)	0.708
	Female	*N* (%)	5 (16.1%)	4 (22.2%)	
History of tobacco use	No	*N* (%)	2 (6.9%)	5 (27.8%)	0.493
	Light	*N* (%)	4 (13.8%)	1 (5.6%)	
	Heavy	*N* (%)	23 (79.3%)	12 (66.7%)	
History of alcohol consumption	No/Social	*N* (%)	19 (67.9%)	13 (72.2%)	1.000
	Heavy	*N* (%)	8 (32.1%)	5 (27.8%)	
Primary tumor site	Oral cavity (including lip)	*N* (%)	10 (32.3%)	12 (66.7%)	0.140
	Oropharynx / Hypopharynx	*N* (%)	4 (12.9%)	1 (5.6%)	
	Larynx	*N* (%)	13 (41.9%)	4 (22.2%)	
	Nose/paranasal sinuses	*N* (%)	4 (12.9%)	1 (5.6%)	
Primary tumor site	Oral cavity	*N* (%)	10 (32.3%)	12 (66.7%)	**0.036**
	Other	*N* (%)	21 (67.7%)	6 (33.3%)	
TNM Stage	I–III	*N* (%)	9 (31.0%)	8 (44.4%)	0.371
	IV	*N* (%)	20 (69.0%)	10 (55.6%)	
Disease setting	Metastatic	*N* (%)	13 (54.2%)	7 (38.9%)	0.252
	Recurrent	*N* (%)	16 (45.8%)	11 (61.1%)	
Line of ICI	1st line	*N* (%)	21 (67.7%)	6 (33.3%)	**0.036**
	2nd–3rd line	*N* (%)	10 (32.3%)	12 (66.7%)	
PD in <3months	No	*N* (%)	22 (71%)	2 (11.1%)	**<0.001**
	Yes	*N* (%)	9 (29%)	16 (88.9%)	
OS (*N* = 49)		Median (95%CI)	11.8 (10–17.7)	5.6 (3.8–11.6)	**0.011**
PFS-immuno (*N* = 49)		Median (95%CI)	4.6 (2.6–6.7)	2.0 (1.7–2.6)	**<0.001**
LDH (*N* = 49)		Median (95%CI)	177 (147–252)	203 (181–270)	0.276
